# Drought-Induced Changes in The Flowering Capacity, Anthesis Quality and Seed Set in Rice (*Oryza sativa* L.)

**DOI:** 10.21315/tlsr2022.33.2.11

**Published:** 2022-07-15

**Authors:** Mohd Syahmi Salleh, Mohd Shukor Nordin, Adam Puteh, Rozilawati Shahari, Zarina Zainuddin, Mohamad Bahagia Ab-Ghaffar, Noraziyah Abd Aziz Shamsudin

**Affiliations:** 1 Department of Plant Science, Kulliyyah of Science, International Islamic University Malaysia, 25200 Kuantan, Pahang, Malaysia; 2 Department of Crop Science, Faculty of Agriculture, Universiti Putra Malaysia, 43400 Serdang, Selangor, Malaysia; 3 Industrial Crop Research Centre, Malaysian Agricultural Research and Development Institute, 13200 Kepala Batas, Pulau Pinang, Malaysia; 4 Department of Biological Sciences and Biotechnology, Faculty of Science and Technology, Universiti Kebangsaan Malaysia, 43600 Bangi, Selangor, Malaysia

**Keywords:** Reproductive Stage Drought, Spikelet Moisture Content, Yield and Its Components, Kemarau Fasa Reproduktif, Kandungan Lembapan Spikelet, Hasil dan Komponennya

## Abstract

Drought stress significantly reduces grain yield (GY) due to poor spikelet fertility and anthesis quality. Aim of this study was to understand the changes of flowering capacity, anthesis quality traits and seed set in the re-watered drought stressed modern high yielding drought susceptible rice cultivar, IR64 at heading (DSH) and booting (DSB) stages. The well-watered plants served as control of the experiment. Results obtained suggest that spikelet moisture content at above 80% was required to maintain optimum anthesis process in rice. Anthesis process in DSH plant was suspended when leaf relative water content (LRWC) dropped to below than 70%. Effects of drought stress on the spikelet moisture were irreversible as compared to the leaf rolling and LRWC. Hence, seed set was failed to occur at the upper rachis branches of the DSH plant. Anthesis process in the re-watered drought stress plants was resumed on the third day after re-watering with about 50% and 80% of anthers managed to dehisce in the DSH and DSB plants. Consequently, percentage of spikelet fertility and seed set in the DSH and DSB plants were increased towards the lower parts of the panicle. The GY, number of seeds, spikelet fertility, and harvest index however were significantly lower in the DSH plant (0.30 g, 13, 16.40% and 14.81) as compared to DSB plant (1.34 g, 57, 59.14% and 48.30), respectively. In addition, all interrelated traits involved in the flowering process of rice could be collectively termed as the anthesis quality traits due to their significant correlation with the grain yield and other yield components.

## Abstract (ms)

Tekanan kemarau mengurangkan hasil bijian (GY) secara signifikan kerana tahap kesuburan spikelet dan kualiti pendebungaan yang rendah. Kajian ini bertujuan untuk memahami dinamika kapasiti pendebungaan, kualiti antesis dan pembentukan biji pada pokok padi yang diberi pengairan semula semasa tekanan kemarau pada dua fasa reproduktif iaitu semasa ‘heading’ (DSH) dan bunting (DSB). Pokok yang tidak diberi tekanan kemarau berfungsi sebagai eksperimen kawalan. Hasil kajian ini menunjukkan bahawa kadar kelembapan spikelet di atas 80% adalah diperlukan untuk proses pendebungaan yang optimum berlaku. Proses pendebungaan oleh pokok DSH terhenti apabila kadar kandungan air relatif daun (LRWC) lebih rendah daripada 70%. Kesan tekanan kemarau terhadap kadar kelembapan spikelet adalah tidak dapat dipulihkan berbanding dengan kadar penggulungan daun dan LRWC. Akibatnya, pembentukan biji padi tidak berlaku di bahagian atas panikel pokok DSH. Proses pendebungaan didapati berlaku pada hari ketiga selepas siraman dilakukan terhadap pokok DSH and DSB dengan kadar 50% dan 80% cepu debunga berjaya mengeluarkan debunga. Kesannya, peratusan kesuburan spikelet dan pembentukan biji oleh pokok DSH dan DSB meningkat pada bahagian bawah panikel. Walaubagaimanapun, GY, jumlah biji, tahap kesuburan spikelet, dan kadar indeks tuaian oleh pokok DSH jauh lebih rendah dan signifikan (0.30 g, 13, 16.40% dan 14.81) berbanding dengan pokok DSB (1.34 g, 57, 59.14% dan 48.30). Sebagai tambahan, semua ciri yang terlibat di dalam proses pendebungaan padi boleh disarankan untuk dinamakan secara kolektif sebagai ciri kualiti antesis kerana hubungan yang signifikan dengan GY dan komponen hasil lain.

**Highlights**

- This study suggests that spikelet moisture content (SMC) at above 80% is required to maintain optimum anthesis process in rice for higher spikelet fertility (SF) and grain yield (GY).
- The efficiency of direct selection for GY under drought might be enhanced by standardising the evaluation and selection of segregating breeding populations at similar reproductive stage particularly at heading prior to the application of life-saving irrigation during field screening.
- All interrelated traits involved in the flowering process of rice mainly spikelet quality, anther dehiscence quality, and pollen quality could be collectively termed as anthesis quality traits due to their significant correlation with the GY and other yield components in rice.

## INTRODUCTION

Rice (*Oryza sativa* L.) is one of the staple foods in the world after wheat and maize (Mishra *et al*. 2019). Unfortunately, rapid increase in the world population has been coupled with the climate change phenomenon such as El-Nino which resulted on challenges in food security (Ikmal *et al*. 2018; Mdluli *et al*. 2020). Drought is one of the most severe climate-related risks for rice production especially in the lowland rainfed areas (Shamsudin *et al*. 2016). This could be due to high susceptibility of lowland semi-dwarf rice cultivars to drought stress. The susceptibility of lowland rice cultivars to drought stress is extremely critical at its reproductive stages ranging from booting to heading (Salleh *et al*. 2018). The occurrence of drought stress during these stages would hamper the production of numerous and viable pollen grains for successful pollination and fertilisation processes in rice.

In addition, failure of anther dehiscence was also reported to be responsible for low spikelet fertility in drought stressed rice at pre-anthesis stage (Liu *et al*. 2006). Failure of anther dehiscence would certainly prevent stigma from receiving pollen grains for the pollination process (Win *et al*. 2020). Above all, spikelet opening during flowering was a prerequisite for anthesis process to occur in rice (Ekanayake *et al*. 1993). Failure of spikelet opening due to severe desiccation caused by drought stress would lead to the failure of anther exertion and dehiscence thus inhibit pollination and fertilisation processes. There are various drought adaptation mechanisms that could be exploited for breeding drought tolerant rice such as drought escape, drought avoidance, drought tolerance and drought recovery (Fukai & Cooper 1995). However, most of the current achievements in breeding drought tolerant rice have been associated with early flowering characteristic of the breeding lines indicating drought escape mechanism (Vikram *et al*. 2016; Shamsudin *et al*. 2016b; Swamy *et al*. 2017) and root traits improvement indicating drought avoidance mechanism (Uga *et al*. 2013; Henry *et al*. 2015; Grondin *et al*. 2018, Ikmal *et al*. 2019).

Nevertheless, in order to further improve yield advantage through drought tolerance mechanism particularly the ability to maintain high water status and delays in typical symptoms of water deficit during severe stress, breeding population should be evaluated at the same phenology group or growth stages (Fukai & Cooper 1995) especially at the most devastating reproductive stage as possible. Conceptually, this kind of approach would reveal the most potential lines having the greatest tolerant ability in maintaining high water status under severe stress condition. However, standard protocol of the widely used breeding method of direct selection for grain yield under drought requires life-saving irrigation practices (re-watering) when soil water potential reached below than −60 kPa (Venuprasad *et al*. 2011). The life-saving irrigation practices however may lead to reversible effects of re-watering on the flowering capacity of the segregating breeding populations.

Hence, the present study was conducted with an aim to attain general understanding on the dynamics of flowering capacity and anthesis quality traits in the high-yielding mega variety IR64 in response to re-watering under reproductive stage drought (RDS) at booting and heading stages. The argument was to prove the hypothesis that re-watering at different reproductive growth stages, i.e., booting and heading stages would result on significantly different responses towards the flowering capacity, anthesis quality traits, harvestable grain yield and other yield components attributes of rice. In addition, the study was also aimed to:

1. Highlight all of the interrelated factors involved in the flowering process of rice such as spikelet quality traits, anther dehiscence quality traits and pollen quality traits which could be collectively termed as anthesis quality traits.
2. Demonstrate that those anthesis quality traits are highly correlated with GY and its components under RDS.

## MATERIALS AND METHODS

### Plant Material

Seeds of rice mega variety, IR64 were originally obtained from the Malaysian Rice Gene Bank, Malaysian Agricultural Research and Development Institute (MARDI) Seberang Perai, Pulau Pinang. According to Mackill and Khush (2018), IR64 is considered susceptible to drought especially reproductive stage drought. The seeds were then selfing for one generation for seed multiplication purposes. The experiment was conducted under glasshouse conditions at the Glasshouse and Nursery Complex (GNC), Kulliyyah of Science, International Islamic University Malaysia (IIUM).

### Experimental Design and Drought Stress Treatment

**Figure 1:**
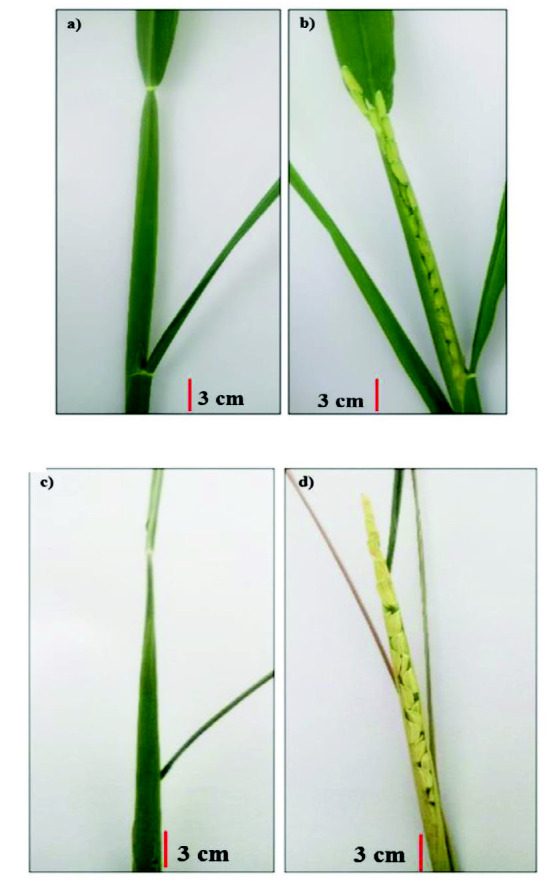

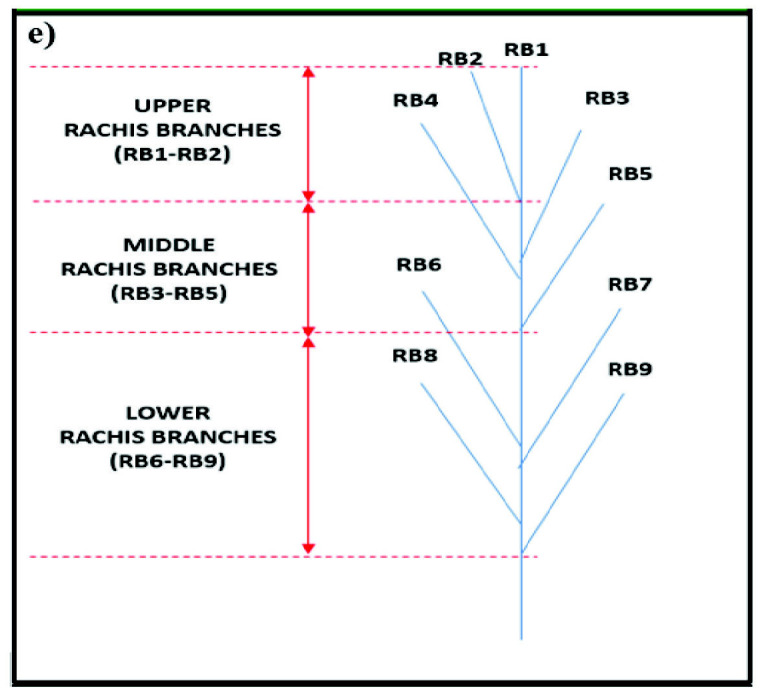
Different stage of rice at reproductive stage: a) Booting stage under well-watered condition, b) Heading stage under well-watered condition, c) Booting stage under drought stressed condition, d) Heading stage under drought stressed condition, and e) Schematic diagram of the location and numbering of rachis branches in tagged panicle.

The experiment was conducted from July to November 2018 using randomised complete block design (RCBD) with three replications. Treatments involved were the T1: well-watered (WW), T2: drought stress at booting (DSB), and T3: drought stress at heading (DSH). The rice panicles have been individually tagged (Fig. 1) and imposed with drought stress based on their reproductive growth stages particularly at booting (about 55 days after planting) and heading (about 60 days after planting) before re-watering on day six after drought stress when soil water potential reached below than −60 kPa (severe drought).

Procedures for RDS was based on the previous study by Salleh *et al*. (2018). Drought stress was imposed by withholding water application. The soil water potential (*ψ*_soil_) was monitored using soil tensiometer (Steizner, GmbH). Water application was resumed when soil water potential reached the desired level of drought stress at −60 kPa (severe stress). Control of the experiment was left under normal level of soil water potential (*ψ*_soil_ = 0 kPa) throughout the study period. The leaf relative water content (LRWC) was also monitored during drought stress treatment and at the early stages of re-watering. The leaf blade was cut and the fresh weight was immediately recorded. Next, the leaf blade was soaked in distilled water for 4 h at 20°C and then blotted dry for turgid weight measurement. The dry weight was obtained after oven-dried for 48 h at 70°C. The LRWC was then calculated based on the equation by Schonfeld *et al*. (1988):


$$LRWC=\frac{fresh\;weight-dry\;weight}{turgid\;weight-dry\;weight}\times 100$$

### Planting Procedures and Agronomic Practices

Plastic trays of size 51 cm × 39 cm × 15 cm containing about 30 kg of silty clay soil (54.4% clay, 44.2% silt and 1.4% sand) were used following Salleh *et al*. (2018). Direct sowing method as normally practiced by most of the local rice farmers was used. All trays were kept under saturated condition before sowing. Twelve pre-germinated seeds were sown in each tray and nine plants were retained after two weeks. Water level was slowly increased based on seedling growth. Finally, standing water at about 3 ± 1 cm was maintained in each tray throughout planting duration except during RDS treatment. Fertilisers were applied based on the planting guide for rice by the Malaysian Agricultural Research and Development Institute (MARDI) (MARDI 2008). Temperature and relative humidity were also recorded using built-in probe. The average value of temperature was at 31.12°C with minimum-maximum values at 24.85°C and 37.38°C while the average value of relative humidity was recorded at 70.38% with minimum-maximum values at 55.71% and 85.04% throughout the experimental period.

### Data Collection

Anthesis quality traits mainly spikelet opening (SO), anther exertion (AE), percentage of anther dehiscence (AD), anther apical dehiscence length (ADL), apical dehiscence width (ADW), basal dehiscence length (BDL), basal dehiscence width (BDW), number of pollen grains per anther (PN), rate of pollen viability (PV), and pollen load on stigma (PLS) were observed and recorded using stereomicroscope with attached camera for four consecutive days starting from the day under severe drought (−60 kPa) until the third days after re-watering. Five samples per replication per treatment were collected for each parameter. Mature anthers from spikelets were collected about one hour before anthesis. Collected anthers were placed in a drop of 1% Iodine Potassium Iodide (IKI) solution on a glass slide and squashed with a narrow scalpel to disperse the pollen grains. The number of pollen grains were observed under stereomicroscope and counted using the ImageJ software. The percentage of viable and sterile pollen grains were counted based on the IKI staining pattern with the dark red coloured (viable) and non-coloured (non-viable) following Prasad *et al*. (2006). Percentage of pollen viability (PV) was then calculated using the following formula:


$$Pollen\;viability(\%)=\frac{number\;of\;viable\;pollen\;grains}{total\;number\;of\;pollen\;grains}\times 100$$

Next, spikelets located at the upper rachis branches of panicle were collected during anthesis and immediately observed under stereomicroscope. The length of SO was measured as a distance between lemma and palea opening. Anther exsertion (AE) was measured based on the length of filament exserted from the spikelet. Anther dehiscence quality (i.e., ADL, ADW, BDL and BDW) were measured based on the length and width of anther opening at the apical and basal part of the thecae following Matsui *et al*. (2005). In addition, about 15 spikelets were collected and immediately weight during anthesis period (between 10.00 am to 12.00 pm) for the determination of spikelet fresh weight (SFW). Collected spikelets were then oven-dried at 70°C for 48 h.

The percentage of spikelet moisture content (SMC) was computed based on the following formula:


$$SMC(\%)=\frac{fresh\;weight-dry\;weight}{fresh\;weight}\times 100$$

For the determination of pollen load on stigma (PLS), spikelets were collected about 1 h after anthesis (after lemma and palea closed). Next, the stigma was excised from individual spikelet and number of pollen grains deposited on the stigma was counted under the microscope after staining with 1% IKI.

The agronomic performance attributes mainly grain yield (GY), number of seed (SN), spikelet fertility (SF), and harvest index (HI) were recorded per panicle basis based on tagged panicle at harvest maturity. Harvested panicles with intact seeds were oven-dried at 50°C until seed moisture content reached about 14 ± 1%. The panicle was divided into three sections namely upper rachis branches (the two topmost branches), middle rachis branches (three branches located immediately below the two topmost branches), and lower rachis branches (the remaining branches located below the middle rachis branches) in order to obtain detailed information on the distribution of seed set and spikelet fertility (Fig. 1). The SF was computed based on the ratio of filled grains or fertile spikelets to the total number of reproductive spikelets following Liu *et al*. (2006):


$$Spikelet\;fertility(\%)=\frac{Total\;number\;of\;fertile\;spikelets\;per\;panicle}{Total\;number\;of\;spikelets\;per\;panicle}\times 100$$

The HI was computed by dividing total grain yield (GY) with total biological yield based on the following formula:


$$Harvest\;index(HI)=\frac{Grain\;weight(g)}{Total\;above\;ground\;biomass(g)}$$

### Statistical Analysis

All data collected were analysed using Analysis of Variance (ANOVA) at *p* ≤ 0.05, Duncan New Multiple Range Test (DNMRT) and Pearson’s correlation analysis using the Statistical Analysis System (SAS) following Salleh *et al*. (2020).

## RESULTS

### Soil Water Potential, Leaf Relative Water Content and Spikelet Moisture Content Response to Drought Stress and Re-watering

**Figure 2:**
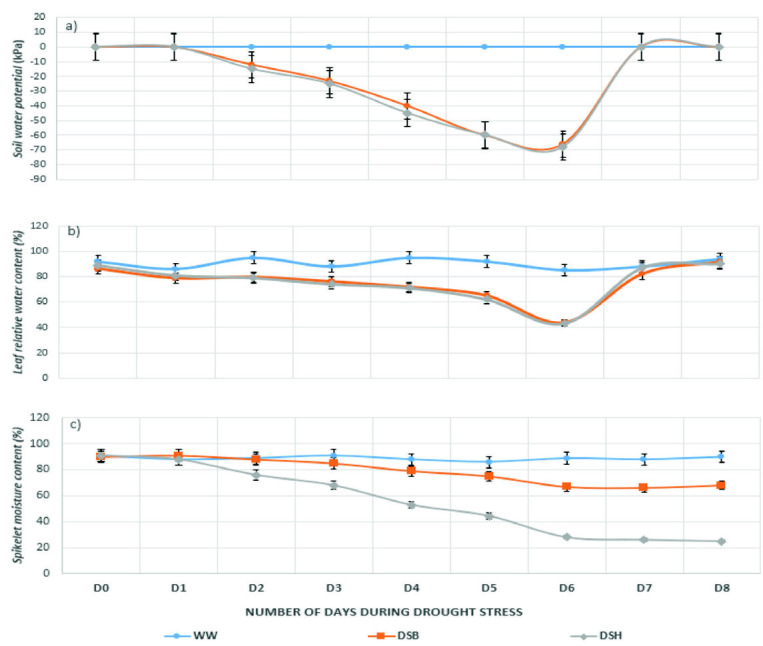
Dynamics of soil water potential, leaf relative water content, and spikelet moisture content. Error bar at each data point is the standard error of the mean: a) Soil water potential; b) Leaf relative water content; and c) Spikelet moisture content at upper rachis branches during drought stress. D0 until D8 indicate number of days during drought stress treatment. Drought stressed plant was re-watered after D6. WW = well-watered, DSB = drought stressed at booting, and DSH = drought stress at heading.

The leaf relative water content (LRWC) dropped to below than 80% when soil water potential reached about −40 kPa on the fourth day after drought stress treatment (Fig. 2). The flag leaf rolling was observed on the fifth day of RDS treatments when the level of soil water potential reached about −60 kPa and the LRWC dropped to below than 70%. On the day six of drought stress treatment, LRWC further dropped to between 35%–50% and soil water potential reached below −60 kPa. All drought stress plants at DSB and DSH were then re-watered since the targeted level of drought stress has been achieved. The soil water potential was restored to normal level at 0 kPa on the next day after re-watering. The flag leaf of DSB and DSH plants however were returned to normal turgid condition at above 80% on the second day after re-watering. This indicated that effects of drought stress on leaf rolling and LRWC was reversible but at a slower rate compared to soil water potential. In contrast, SMC of the DSH plant remains at below than 30% even after re-watering.

### Changes of Flowering Capacity and Anthesis Quality Traits in Response to Drought Stress and Re-watering

As a result of severe desiccation, anthesis of dried spikelets located at the upper rachis branches of DSH plant was inhibited (Table 1). Nevertheless, heading process (the emergence of new spikelets from the flag leaf sheath) in the DSH plant was resumed on a subsequent day (first day after re-watering). However, the newly emerged spikelets located at the middle rachis branches were partially dried and remains un-open throughout the day. On the second day after re-watering, the previously emerged spikelets were observed to be opened. However, failure of anther dehiscence in the DSH plant was observed in the opened spikelets with all anthers remains intact even after lemma and palea closed. Pollination process was prohibited due to failure of anther dehiscence and pollen release. Surprisingly, the rate of pollen viability in those failed to dehisce anthers in the DSH plants was found to be high at about 96% as shown in Table 1. The value, however, was significantly lower as compared to the well-watered plants. On the third day after re-watering, anthesis in the DSH plant successfully occurred with about 50% anthers of opened spikelets managed to dehisce. However, the rate of PLS in the DSH plant was significantly lower as compared to the well-watered plant. This might be due to low amount of PN and rate of dehisced anthers. In the case of DSB plants, heading process was resumed on the second day after re-watering. The exserted spikelets however were partially dried at the spikelet moisture content of about 60%. Anthesis successfully occurred on the third day after re-watering with about 80% of anthers manage to dehisce (Table 1). The number of PN and PLS in the DSB plant was significantly lower as compared to WW plants but slightly higher than the DSH plant.

### Agronomic Performance

Well-watered plants recorded significantly higher GY at 2.08 g and yield components attributes such as SN at 81 seeds, SF at 92.87%, and HI at 0.60 as compared to both DSH and DSB plants (Table 2). The SF, SN, GY, and HI were significantly lower in the DSH plants (16.40%, 13 seeds, 0.30 g and 0.15) as compared to the DSB plants (59.14%, 57 seeds, 1.34 g and 0.48). However, the GY reduction in DSH plant was recorded at about 86% while the DSB plant only recorded about 36% reduction as compared to the WW plant. This result suggests that although the DSH and DSB plants were similarly stressed at soil water potential lower than −60 kPa and LRWC level of between 35%–50%, the effects on GY reduction during DSH was found to be more severe as compared to DSB (based on GY reduction).

In addition, SF in DSH plants was increased towards the lower parts of the panicle. The re-watering SF of the DSH plant to about 14% for the middle rachis branches (SFM) and 33% for the lower rachis branches (SFL). Consequently, the SN in the DSH plant was also increased towards the lower parts of the panicle. In the well-watered plant, the trend of seed set was also increased from top to bottom parts of the panicle. Total SF in the well-watered plant was about 93% with almost equal distribution of SF across the panicle ranging from 90 to 94% (Table 2). Similar trend was observed in the DSB plants although SF was significantly lower as compared to WW plants ranging from 57% to 61% across the panicle (Table 2).

### Correlation Analysis

In general, the GY, SF, and SN were significantly correlated with all of the anthesis quality traits (Table 3). The HI was also significantly correlated with all of the anthesis quality traits except for the anther BDL and BDW. The SF of upper rachis branches (SFU), middle rachis branches (SFM), and lower rachis branches (SFL) were also significantly correlated with all of the anthesis quality traits. Similar observation was also recorded in the number of seeds at the lower rachis branches (SNL). The number of seeds at the upper rachis branches (SNU) was also significantly correlated with all of the anthesis quality traits except for the anther BDW. In contrast, number of seeds at the middle rachis branches (SNM) was not significantly correlated with all of the anthesis quality traits except for the PV. Result of the correlation analysis indicated that spikelet quality attributes (SO and SMC), anther dehiscence quality attributes (AE, AD, ADL, ADW, BDL and BDW), and pollen quality attributes (PN, PV and PLS) were significantly correlated with the GY and other yield components suggesting that those flowering traits could be collectively termed as the anthesis quality traits.

## DISCUSSION

Reproductive stage drought (RSD) significantly reduced GY due to lower percentage of filled spikelet cause by poor anthesis quality. Previous studies by Salleh *et al*. (2021), Ikmal *et al*. (2019), Salleh *et al*. (2018), and Shamsudin *et al*. (2016a; 2016b) indicated that GY were significantly reduced under RSD. However, those studies do not clearly explain the mechanism and changes in the flowering capacity, anthesis quality, and seed set in rice due to RSD and re-watering activity particularly the life-saving irrigation practices during field selection in the method of direction for GY under drought. In fact, most of previous studies conducted were focusing on the response of leaf related traits towards drought mainly the leaf relative water content (RWC) (Gupta *et al*. 2020), leaf anatomy and ultra-structure (Upadhyaya & Panda 2019), and the leaf growth, rate of photosynthesis and other metabolic processes (Zhu *et al*. 2020).

The present study hence was conducted to understand the physiological changes occurred in the flowering trait of rice due to re-watering activity during RSD. Detailed information on the response of anthesis quality traits to RSD may provide better understanding on the reversible and irreversible effects of re-watering on the rice flowering traits. In more specific, the present study compared the effects of re-watering on drought stressed rice at two reproductive growth stages (DSB and DSH) in the high-yielding mega variety IR64 yet drought susceptible. Briefly, SO was completely prevented at the upper rachis branches of DSH plants under severe drought stress condition (Table 1). This could be due to the irreversible effects of desiccation (drought stress) on the spikelet moisture content of the DSH plants (Fig. 2). The level of SMC after re-watering remains at the same level as under drought stress condition (below 30%) albeit LRWC was restored to the normal level at more than 80%. According to Hsiao (1973), plants are considered to be under stress when the LRWC dropped to below than 80%. The irreversible effects of drought stress on SMC has led to complete (100%) spikelet sterility and failure of seed set at the upper rachis branches of DSH plants. Similar observation was reported by Ekanayake *et al*. (1989) due to the fact that rice spikelet does not have a dynamic water conservation mechanism like the leaf. Hence, rice genotype having superior ability in maintaining higher SMC during severe drought stress would certainly possess drought tolerance ability. Jongdee *et al*. (2002) which contrasted in maintenance of leaf water potential (LWP and Mishra *et al*. 2019) also reported that rice genotypes and breeding lines with the ability to maintain high water potential, good osmotic adjustment, photosynthetic rate, transpiration rate, and stomatal conductance during severe drought stress under field condition would certainly have higher tolerance ability.

In the case of AD, the present study recorded similar observation as reported by Liu *et al*. (2006) that basal part of anthers of both DSB and DSH plants failed to dehisce after 15 minutes of spikelet opening. As result, pollen grains were not totally released from the anther leading to a lower PN deposited on the stigmatic surface. This could be among a reason for significantly lower SF, SN, GY and HI in the DSB and DSH plants as compared to WW plants. Sanders *et al*. (2000) and Liu *et al*. (2006) also stated that programmed cell death of the endothecium cells located at the apical and basal part of an anther was responsible for the anther dehiscence process and subsequent release of pollen grains. Irreversible effects of drought stress on anther proteome of susceptible genotype IR64 may be responsible in reducing the efficiency of programmed cell death of the endothecium cells located at the basal part of the anther for anther dehiscence and pollen release processes (Liu & Bennett 2011).

In addition, PN deposited on stigma and speed of pollen tube growth would also influence the success rate of fertilisation. Conceptually, only one pollen grain is needed to fertilise the egg. However, in rice, a minimum number of about 10 to 20 germinated pollen grains on the stigmatic surface are required for fertilisation process to be successfully occurred due to the nature of cooperation among pollen grains (Matsui & Kagata 2003; Liu *et al*. 2006; Jagadish *et al*. 2011). In the present study, the PN, PV and PLS were significantly reduced in drought stress plants. These findings were corroborated with previous studies by Liu *et al*. (2006), Jagadish *et al*. (2011), and Saragih *et al*. (2013) which reported that the PN, PV and PLS in drought stressed plants were significantly lower as compared to WW plants based on the IKI staining pattern.

Surprisingly, effects of drought stress on PV were found to be reversible by the re-watering activity. As evidence, the PV in both DSH and DSB plants were not significantly different with WW plants on the third day after re-watering (Table 1). Therefore, it could be postulated that drought stress treatment in the present study did not disrupt normal process of starch accumulation in the pollen grains of spikelets located at the lower rachis branches. Water potential of pollen grains in those lower rachis branches could also be regarded as normal condition. This could be due that those spikelets were still enclosed inside the sheath of flag leaf during drought stress period. Consequently, SMC at the lower rachis branches was maintained at above severe desiccation level, unlike the upper and middle rachis branches spikelets. Hence, the process of spikelet development along with microsporogenesis and microgametogenesis in those anthers at the lower rachis branches may normally occur due to higher spikelet and anther moisture content resulted in higher rate of PV in those anthers.

**Figure 3:**
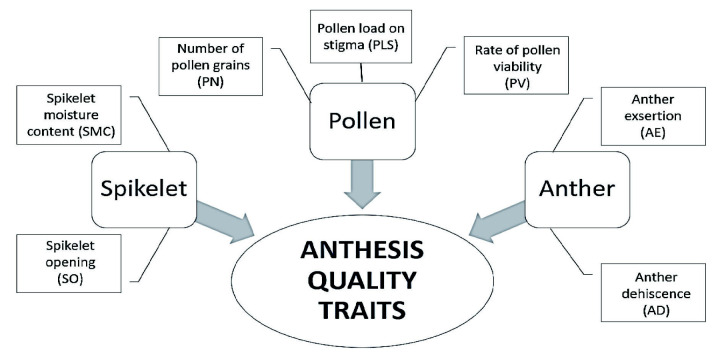
The anthesis quality traits: all interrelated traits involved in the flowering process of rice particularly spikelet quality, anther dehiscence quality, and pollen quality which significantly correlated with the grain yield and other yield components.

In overall, DSH cause higher severity on GY reduction as compared to at DSB which only recorded moderate GY reduction. Total spikelet fertility, GY and HI were also significantly lower in the DSH plant (16.40%, 0.30 g and 14.81) as compared to the DSB plant (59.14%, 1.34 g and 48.30) and well-watered (WW) plants (92.87%, 2.08 g and 60.67), respectively. These results supported hypothesis of the present study that re-watering activity at different reproductive growth stages particularly at booting and heading stages resulted on different responses towards the flowering capacity, anthesis quality traits, harvestable grain yield and other yield components attributes of rice. Hitherto, Fukai and Cooper (1995) suggested that genotypes should be compared for drought resistance or susceptibility within the same phenology group and growth stages. Therefore, it could be suggested that selection being made on the breeding lines at heading stage before the life-saving irrigation practices during field selection in the method of direct selection for GY under drought might reveal the most potential lines having greater drought tolerance ability in maintaining superior anthesis quality traits and high yield components attributes under severe stress. In addition, this approach may also assist breeders in reducing the size of breeding population at early reproductive stage and advancing only highly potential lines for further selection. On the other notes, Yang *et al*. (2019) suggested that high recovery ability of rice plants towards drought and re-watering activity at flowering stage may be useful for plant breeder as one of the selection criterions in breeding drought tolerant rice. Results of the present study also indicated that most of the anthesis quality traits were significantly correlated with the GY and its components (Table 3). This indicated that those traits were jointly involved in the anthesis of rice ensued with higher harvestable GY and yield components. Based on this avenue, the present study proposed that those interrelated traits be collectively termed as anthesis quality traits (Fig. 3). To date, the term anthesis quality traits have yet to be introduced in the rice research.

## CONCLUSION

In conclusion, DSH caused severe GY reduction as compared to at DSB. Anthesis at heading was suspended when LRWC dropped to below 70%. This study suggested that SMC at above 80% was required to maintain optimum anthesis in rice for higher SF and GY. The efficiency of direct selection for GY under drought might be enhanced by standardising the evaluation and selection of segregating breeding populations at similar reproductive stage particularly at heading before the application of life-saving irrigation during field screening. In addition, all interrelated traits involved in the flowering process of rice (i.e., spikelet quality, anther dehiscence quality, and pollen quality) could be collectively termed as anthesis quality traits due to their significant correlation with the GY and other yield components.

## Figures and Tables

**Table 1:** Dynamics of anthesis quality traits in the re-watered drought stress rice.

Trait	Treatment	WW	DSB	DSH
SO (μm)	*Severe drought stress*	2140.44^a^ (±25.10)	0.00^b^ (±0.00)	0.00^b^ (±0.00)
AE (μm)		5054.30^a^ (±134.25)	0.00^b^ (±0.00)	0.00^b^ (±0.00)
AD (%)		100^a^ (±0.00)	0.00^b^ (±0.00)	0.00^b^ (±0.00)
ADL (μm)		330.22^a^ (±34.06)	0.00^b^ (±0.00)	0.00^b^ (±0.00)
ADW (μm)		148.58^a^ (±10.82)	0.00^b^ (±0.00)	0.00^b^ (±0.00)
BDL (μm)		323.62^a^ (±82.38)	0.00^b^ (±0.00)	0.00^b^ (±0.00)
BDW (μm)		146.01^a^ (±48.66)	0.00^b^ (±0.00)	0.00^b^ (±0.00)
PN		2211.00^a^ (±75.41)	848.67^b^ (±49.88)	786.33^b^ (±35.67)
PV (%)		98.67^a^ (±0.3)	97.33^a^ (±0.3)	91.67^b^ (±0.8)
PLS		46.33^a^ (±13.9)	0.00^b^ (±0.00)	0.00^b^ (±0.00)

SO (μm)	*First day after re-watering*	2071.98^a^ (±266.7)	0.00^b^ (±0.00)	0.00^b^ (±0.00)
AE (μm)		4466.74^a^ (±74.05)	0.00^b^ (±0.00)	0.00^b^ (±0.00)
AD (%)		100^a^ (±0.00)	0.00^b^ (±0.00)	0.00^b^ (±0.00)
ADL (μm)		359.86^a^ (±23.55)	0.00^b^ (±0.00)	0.00^b^ (±0.00)
ADW (μm)		156.02^a^ (±26.63)	0.00 ^b^ (±0.00)	0.00 ^b^ (±0.00)
BDL (μm)		195.98^a^ (±7.84)	0.00^b^ (±0.00)	0.00^b^ (±0.00)
BDW (μm)		67.45^a^ (±6.64)	0.00^b^ (±0.00)	0.00^b^ (±0.00)
PN		2254.00^a^ (±142.12)	955.00^b^ (±49.54)	893.30^b^ (±22.88)
PV (%)		98.33^a^ (±0.3)	97.0^ab^ (±0.6)	94.33^b^ (±0.8)
PLS		52.00^a^ (±10.5)	0.00^b^ (±0.00)	0.00^b^ (±0.00)

SO (μm)	*Second day after re-watering*	2057.40^a^ (±77.15)	0.00^b^ (±0.00)	1622.60^a^ (±99.15)
AE (μm)		4184.20^a^ (±200.50)	0.00^b^ (±0.00)	4233.51^a^ (±320.13)
AD (%)		100^a^ (±0.00)	0.00^b^ (±0.00)	0.00^b^ (±0.00)
ADL (μm)		407.77^a^ (±19.61)	0.00^b^ (±0.00)	0.00^b^ (±0.00)
ADW (μm)		153.30^a^ (±14.58)	0.00^b^ (±0.00)	0.00^b^ (±0.00)
BDL (μm)		392.63^a^ (±60.38)	0.00^b^ (±0.00)	0.00^b^ (±0.00)
BDW (μm)		125.16^a^ (±29.22)	0.00^b^ (±0.00)	0.00^b^ (±0.00)
PN		2220.0^a^ (±190.42)	1212.3^b^ (±87.74)	901.00^b^ (±36.51)
PV (%)		98.67^a^ (±0.3)	97.00^b^ (±0.6)	96.00^b^ (±0.6)
PLS		57.00^a^ (±10.4)	0.00^b^ (±0.00)	0.00^b^ (±0.00)

SO (μm)	*Third day after re-watering*	2216.80^a^ (±199.0)	2080.00^a^ (±86.83)	1821.50^a^ (±107.2)
AE (μm)		3931.60^a^ (±100.03)	2797.00^b^ (±272.91)	2514.00^b^ (±295.18)
AD (%)		100^a^ (±0.00)	77.67^a^ (±5.33)	50.00^b^ (±9.81)
ADL (μm)		339.25^a^ (±53.80)	286.63^a^ (±32.10)	319.43^a^ (±28.29)
ADW (μm)		138.35^a^ (±24.14)	120.08^a^ (±3.36)	91.57^a^ (±24.70)
BDL (μm)		313.38^a^ (±62.71)	0.00^b^ (±0.00)	0.00^b^ (±0.00)
BDW (μm)		112.38^a^ (±35.23)	0.00^b^ (±0.00)	0.00^b^ (±0.00)
PN		2100.67^a^ (±26.03)	1412.33^b^ (±25.67)	903.00^c^ (±43.14)
PV (%)		98.67^a^ (±0.3)	98.67^a^ (±0.3)	98.00^a^ (±0.6)
PLS		64.33^a^ (±13.5)	23.67^b^ (±4.81)	8.67^b^ (±2.33)

*Notes*:

* Values in the same column with different superscripts alphabet are significantly different at *p* ≤ 0.05.

Values in *parenthesis are the standard error of the mean*. WW = Well-watered, DSB = Drought stress at booting, DSH = Drought stress at heading, SO = Spikelet opening, AE = Anther exsertion, AD = Anther dehiscence, ADL = Apical dehiscence length, ADW = Apical dehiscence width, BDL = Basal dehiscence length, BDW = Basal dehiscence width, PN = Pollen number, PV = Pollen viability, and PLS = Pollen load on stigma.

**Table 2:** Effects of pre-anthesis drought stress on yield components attributes.

	SF (%)	SFU (%)	SFM (%)	SFL (%)	SN	SNU	SNM	SNL	GY (g)	HI
WW	92.87^a^ (±0.56)	94.34^a^ (±0.96)	90.80^a^ (±1.36)	93.47^a^ (±1.05)	81^a^ (±0.88)	20^a^ (±1.45)	25^a^ (±1.20)	35^a^ (±1.53)	2.08^a^ (±0.03)	0.60^a^ (±0.02)
DSB	59.14^b^ (±1.94)	59.34^b^ (±5.16)	57.15^b^ (±1.98)	60.93^b^ (±2.92)	57^b^ (±1.45)	11^b^ (±2.03)	23^a^ (±1.76)	22^b^ (±0.33)	1.34^b^ (±0.08)	0.48^b^ (±0.01)
DSH	16.40^c^ (±0.56)	0.00^c^ (±0.00)	13.73^c^ (±3.19)	33.24^c^ (±1.14)	13^c^ (±2.73)	0^c^ (±0.00)	5^b^ (±1.20)	8^c^ (±1.53)	0.30^c^ (±0.05)	0.15^c^ (±0.02)

*Notes:*

* Values in the same column with different superscripts alphabet are significantly different at *p* ≤ 0.05.

Values in parenthesis are the standard error of the mean. WW = Well-watered, DSB = Drought stress at booting, DSH = Drought stress at heading, SF = Total spikelet fertility per panicle, SFU = Spikelet fertility of upper branches, SFM = Spikelet fertility of middle branches, SFL = Spikelet fertility of lower branches, SN = Total number of seed per panicle, SNU = Number of seed at upper branches, SNM = Number of seed at middle branches, SNL = Number of seed at lower branches, GY = Grain yield, and HI = Harvest index.

**Table 3:** Correlation analysis of anthesis quality traits and yield components.

	SO	AE	AD	ADL	ADW	BDL	BDW	PN	PV	PLS
SFU	0.78^**^	0.78^**^	0.78^**^	0.76^**^	0.77^**^	0.72*	0.68*	0.79^**^	0.93^***^	0.69*
SFM	0.82^**^	0.82^**^	0.82^**^	0.80^**^	0.82^**^	0.77*	0.73*	0.84^**^	0.91^***^	0.73*
SFL	0.88^**^	0.88^**^	0.88^**^	0.87^**^	0.87^**^	0.80^**^	0.75*	0.90^***^	0.91^***^	0.79^**^
SF	0.83^**^	0.83^**^	0.83^**^	0.81^**^	0.82^**^	0.76*	0.72*	0.85^**^	0.93^***^	0.74*
SNU	0.81^**^	0.81^**^	0.81^**^	0.80^**^	0.79^**^	0.68*	0.63^ns^	0.81^**^	0.89^***^	0.74*
SNM	0.56^ns^	0.56^ns^	0.56^ns^	0.54^ns^	0.56^ns^	0.56^ns^	0.53^ns^	0.59^ns^	0.93^***^	0.51^ns^
SNL	0.84^**^	0.84^**^	0.84^**^	0.84^**^	0.84^**^	0.76*	0.72*	0.85^**^	0.89^***^	0.69*
SN	0.77*	0.77^**^	0.77^**^	0.76^**^	0.77^**^	0.69*	0.66*	0.78^**^	0.94^***^	0.67*
HI	0.70*	0.70*	0.70*	0.68*	0.68*	0.64^ns^	0.60^ns^	0.72*	0.94^***^	0.66*
GY	0.81^**^	0.81^**^	0.81^**^	0.80^**^	0.80^**^	0.75*	0.71*	0.82^**^	0.92^***^	0.71*

*Notes:*

* The ^***^, ^**^ and ^*^ denotes significant correlation at *p* ≤ 0.001, *p* ≤ 0.01 and *p* ≤ 0.05 while ‘^ns^’ denotes not significantly correlated at *p* ≤ 0.05.

SO = Spikelet opening, AE = Anther exsertion, AD = Anther dehiscence, ADL = Apical dehiscence length, ADW = Apical dehiscence width, BDL = Basal dehiscence length, BDW = Basal dehiscence width, PN = Pollen number, PV = Pollen viability, PLS = Pollen load on stigma, SFU = Spikelet fertility of upper branches, SFM = Spikelet fertility of middle branches, SFL = Spikelet fertility of lower branches, SF = Total spikelet fertility per panicle, SNU = Number of seed at upper branches, SNM = Number of seed at middle branches, SNL = Number of seed at lower branches, SN = Total number of seed per panicle, HI = Harvest index, and GY = Grain yield.
